# Periductal Mastitis: An Inflammatory Disease Related to Bacterial Infection and Consequent Immune Responses?

**DOI:** 10.1155/2017/5309081

**Published:** 2017-01-15

**Authors:** Lu Liu, Fei Zhou, Pin Wang, Lixiang Yu, Zhongbing Ma, Yuyang Li, Dezong Gao, Qiang Zhang, Liang Li, Zhigang Yu

**Affiliations:** ^1^Department of Breast Surgery, The Second Hospital of Shandong University, Jinan, Shandong 250033, China; ^2^School of Medicine, Shandong University, Jinan, Shandong 250012, China; ^3^Department of Breast Surgery, The Third People's Hospital of Chengdu, Chengdu, Sichuan 610031, China

## Abstract

Periductal mastitis (PDM) is a prolonged inflammatory disease, but the cause of PDM is poorly understood. In the present case control study, 87 PDM and 87 healthy controls were enrolled and the results were evaluated to identify the significant risk factors for PDM. To investigate the roles of bacterial infection and critical cytokines expression, 16S rRNA gene sequencing and bacterial culturing were conducted. We also measured the levels of interferon-*γ*, interleukin-12A, and interleukin-17A by semiquantitative immunohistochemistry method. In a multivariable logistic regression model, we identified overweight/obesity and late onset of menarche as independent risk factors for PDM. In contrast, age of first birth >27 years had a protective effect. With 16S rRNA gene sequencing, we confirmed bacterial infections were found in all PDM patients, but none of the control patients was positive on the gene expression of 16S rRNA. Our results also demonstrated significant increases of the IFN-*γ* and IL-12A expression in PDM, but there was no difference in IL-17A expression in these two groups. Taken together, this study suggests that reproductive factors and overweight/obesity are possible predisposing risk factors for PDM. Bacterial infection and the increased expression of some proinflammatory cytokines are associated with the pathogenesis of this disease.

## 1. Introduction

Periductal mastitis (PDM) is histopathologically defined as a chronic inflammation of the breast, with dilation of the mammary duct, plasma cell infiltration, and abscess formation. A variety of different terms, including mammary ductal ectasia and plasma cell mastitis, have been used for this condition [1, 2]. The use of different terms probably reflects different stages in the disease process. In recent years, the morbidity rate of PDM has risen rapidly [3]. Moreover, in several studies, mammary ductal ectasia has been found to be responsible for 5–25% of all symptomatic breast conditions [4, 5]. The clinical presentations of PDM are not specific and are easily confused with breast carcinoma in imaging manifestations [6, 7].

Thus far, the etiology of PDM is still unknown. Several factors have been associated with an increased risk for this disease, including smoking, obesity, and diabetes mellitus. However, all of these stated risks are identified based on small case series reports and have not been confirmed epidemiologically [8, 9]. Although PDM is generally seen in those who smoke heavily [9], some other studies also suggest inconsistent findings with this conclusion [10]. In addition, although obesity is considered as a significant risk factor for PDM [11], this conclusion is also just based on case reports, lacking epidemiological evidence. Literature also indicates conflicting information on whether age at first birth, parity, and duration of breastfeeding are potential risk factors for PDM [4, 12]. Moreover, while some studies claim that ethnicity is associated with PDM [13], these studies were limited to a few of geographical areas and races since all data are concluded from small scale studies. Furthermore, as we have known now, the data on the predisposing factors for PDM in Chinese patients is very rare too.

Etiologically, the exact role of bacterial infection in PDM has been debated for many years. Bacterial infection was thought to be a possible etiological factor of PDM. However, the current overall data are not conclusive as some studies also suggest sterile lesions in PDM patients without invasion of any bacteria [14]. Among the studies that demonstrated the presence of bacterial infection, both aerobes and anaerobes have been found in the subsequent culturing or sequencing of samples from PDM patients, including* Pseudomonas aeruginosa* and* Staphylococcus aureus* [12]. Although the diversity of bacterial strains which infected the lesions of PDM might be due to different detecting methods and culture media used in different individual study, the overall results still suggest that a specific pathogenic bacterium may not exist for the infection of PDM [15, 16]. Therefore, we hypothesize that the main cause of this disease may be the consequent immune response following bacterial infection instead of the infection itself. However, there is little data available now on the literature about the role of immune response in this disease. It has been well-established that adaptive immune responses play very important roles in the development of inflammatory diseases, especially in those related to bacterial infection. Key players of the adaptive immune response are T helper (Th) cells [17]. Th cells have been divided into three major subsets based on their different cytokine expression profiles. The Th1 subset is characterized by the production of interferon-*γ* (IFN-*γ*) and IL-12; while the major cytokines featured in the Th2 subset are interleukin-4 (IL-4) and IL-10, respectively. The Th17 subset was described more recently and is simply defined by the production of IL-17. As reported, while Th1 subset controls intracellular bacterial infections, the Th2 subset controls parasitic infections and the Th17 subset controls fungal and extracellular bacterial infections, respectively [18]. Since PDM is a condition related to bacterial infection, it is reasonable to assume that Th1 and Th17 may mediate the possible immune responses following bacterial infection in PDM.

In the present study, besides exploring and identifying the possible risk factors for PDM, we also investigate the relationship between bacterial infection and PDM and the significance of the expression of consequent Th1 and Th17 cytokines in this disease. Our results may help to further elucidate the pathogenesis of PDM. Furthermore, since there are very few well-designed investigations tending to explore the risk factors and possible causes for PDM in Chinese patients now, our results here may lay an important ground for the future epidemiological study on this issue in Chinese Han population.

## 2. Materials and Methods

### 2.1. Study Population

This was a retrospective nonmatched case control study. Eighty-seven patients with PDM were recruited from the Department of Breast Surgery at the Second Hospital of Shandong University. The study was conducted from January 2011 to March 2015. The inclusion criteria for patients with PDM were as follows: (I) newly diagnosed and histologically confirmed periductal mastitis; (II) Han ethnic group; and (III) no evidence or history of cancer. Eighty-seven healthy controls were collected from the Physical Examination Center of the Second Hospital of Shandong University from June 2015 to August 2015. The criteria for the control group were as follows: (I) normal results on breast physical examinations, breast ultrasound scans, and/or mammographic screening and without any possible benign breast diseases (fibroadenoma, PDM, breast cyst, intraductal papillomas, etc.); (II) Han ethnic group; and (III) no evidence or history of breast cancer. All eligible subjects were women in reproductive age and willing to participate in the study voluntarily. All procedures performed here involving human participants were approved by the Second Hospital of Shandong University Research Committee.

### 2.2. Pathological Diagnosis

Vacuum-assisted, incisional, or excisional biopsy was performed based on the clinical findings at the time of admission. Pathological results were reviewed by two pathologists. PDM patients were included in this study only if they had confirmed pathological changes. All patients with other possible causes of mammary inflammation were ruled out, such as breast tuberculosis, fat necrosis, and granulomatous lobular mastitis, as well as inflammation due to lactation or pregnancy.

### 2.3. Data Collection

Possible etiologic factors were written down at the time of admission, including sociodemographic status, smoking status, alcohol consumption, reproductive history, and medical history. In addition, the following information was also obtained: patient age, marital status, menopause status, height, weight, body mass index (BMI), active and passive smoking, alcohol abuse, heart and other chronic disease history, history of allergies, history of benign breast disease, galactostasis, age at menarche, age of first child birth, parity, miscarriage history, and duration of breastfeeding. For individual patient, clinicopathologic characteristics were also collected, including major complaint on presentation, size of lesion, and histology findings.

### 2.4. Bacteria Detection

For the specimen collection, patients who received any antibiotics before admission and had sinus or fistula or abscess incision drainage were ruled out from the study. Finally, 33 specimens from 31 patients, including 21 pus samples and 12 tissue samples, were collected in this study. Normal tissues adjacent to benign breast pathology (including fibroadenoma, mastalgia, and intraductal papilloma of the breast) were used as control specimens (*n* = 12). Breast tissue samples were collected under aseptic conditions at the time of operation. Tissues were divided into two sections, with one section frozen immediately in liquid nitrogen and another section sent to the laboratory for bacterial culture. Fresh breast specimens were homogenized and incubated onto Columbia blood agar (Babio Biotech Co., Ltd., Jinan, China) and anaerobic blood culture bottles (Biotech Co., Ltd.). The agar plates were incubated at 37°C and humidified incubator was supplied with 10% carbon dioxide. The colonies formed in the plates were picked and analyzed using a MicroScan Walkaway-96 system (Dade-Behring MicroScan, Sacramento, CA). Gram staining and a stain for acid-fast bacilli were conducted for all specimens. All samples were also analyzed by 16S rRNA gene sequencing. Genomic DNA was prepared using the bacterial DNA extraction kit (Tiangen Biotech Co., Ltd., Beijing, China) according to the manufacturer's recommendations. The hypervariable regions V1–V3 of the 16S rRNA gene were PCR-amplified using universal primers (V3F: 5′-CCAGACTCCTACGGGAGGCAG-3′, V3R: 5′-CGTATTACCGCGGCTGCTG-3′; 27F: 5′-AGA GTT TGA TCM TGG CTC AG-3′, 519R: 5′-GWA TTA CCG CGG CKG CTG-3′) (Sangon Biotech Co., Ltd., Shanghai, China) [19]. The 203 and 492 bp PCR products were purified after agarose gel electrophoresis and sequences were determined using the ABI PRISM 3730 DNA sequencer (Perkin Elmer Inc., Waltham, MA, USA). Nucleotide sequences were analyzed using the National Center for Biotechnology Information BLAST software (https://www.ncbi.nlm.nih.gov).

### 2.5. Immunohistochemistry

For the patients who were tested for bacterial infection, tissue samples which are mentioned above were also embedded in paraffin (*n* = 31) for immunohistochemistry study of proinflammatory cytokines. Normal tissues adjacent to benign breast pathology (including fibroadenoma, mastalgia, and intraductal papilloma of breast) were used as control specimens (*n* = 22). Immunohistochemistry was performed on 4 *μ*m thick sections. And then, the streptavidin-peroxidase-biotin (SP) immunohistochemical method was performed to detect the expression of IFN-*γ*, IL-12A, and IL-17A. Briefly, after deparaffinization and rehydration, the human tissue sections were incubated in 3% hydrogen peroxide in methanol to quench the endogenous peroxidase activity, followed by incubation with normal goat serum to block nonspecific binding. Samples were then incubated overnight at 4°C with rabbit anti-IFN-*γ* (1 : 500, ab9657, Abcam, Cambridge, MA, USA), anti-IL-12A (1 : 500, ab131039, Abcam), or anti-IL-17A (1 : 500, ab9565, Abcam). The secondary antibody was from the SP reagent kit (PV9000; Zhongshan Biotech Co., Ltd., Beijing, China). The color reaction was performed with 3,3′-Diaminobenzidine (Zhongshan Biotech Co., Ltd.), counterstained with hematoxylin, dehydrated, treated with xylene, and then mounted. For negative controls, the antibodies were replaced with PBS. To determine the ratio of positive cells for these three cytokines, 5 fields (×400) were randomly examined per slide and scored by two pathologists who are blinded to the patients' information. The levels of IFN-*γ*, IL-12A, and IL-17A expression were semiquantitatively expressed using a visual grading system based on the extent of staining (percentage of positive cells graded on scale from 0 to 3: 0, none; 1: 1–30%; 2: 31–60%; 3: >60%) and the intensity of staining (graded on a scale of 0–3: 0, none; 1: weak staining; 2: moderate staining; 3: strong staining). The combination of extent (E) and intensity (I) of staining was obtained by the product of E and I, with EI varying from 0 to 9 for each spot. Low expression was defined as EI ≤ 3, and others were considered high expression [20].

### 2.6. Statistical Analysis

Statistical analysis was performed with SPSS 17.0 software (SPSS Inc., Chicago, IL, USA). The results were reported as counts (percentage) for the categorical variables, and mean ± standard deviation for the continuous variables. A two-sample Student's *t*-test was used to compare the difference between the means of the continuous variables. Chi-square test (or Fisher exact test) was used to compare the differences between the proportions if appropriate. Risk factors for PDM were further assessed using both univariate and multivariable (unconditional) logistic regression models. The continuous variables were evaluated according to the clinically relevant categories. The categories for BMI were based on the criteria of being overweight and obese for Chinese individuals (overweight: 24–27.9 kg/m^2^; obese: ≥28 kg/m^2^) [21]. The categories for other continuous variables were on the basis of references (menarche age ≤ 14 years, >14 years; times of parity ≤ 1, >1; age at birth of first child ≤ 27 years, >27 years; duration of breastfeeding ≤ 12 months, >12 months) [22, 23]. Odds ratio (OR) with 95% confidence interval (95% CI) was used to estimate the association between risk factors and exposure. The significance level was set to *P* < 0.05 (two-tailed).

## 3. Results

### 3.1. Study Population Characteristics

To investigate the risk factors for PDM, 87 patients and 87 healthy control subjects were enrolled in this study. The characteristics of patients and controls are summarized in Table 1. The median age of patients at presentation was 34 (range 20–62) years, and the median age of controls was 34 (range 26–45) years. The median age did not differ significantly between the two groups (*P* = 0.134). The majority of subjects in this study were married. There were no statistically significant differences between these two groups in terms of for the history of hypertension, type 2 diabetes mellitus, heart disease, or any history of allergy. Neither alcohol abuse nor active smoking was reported in the case and control groups. There was not any form of breast cancer in these patients of PDM confirmed by pathologic examination.

**Figure 1:**
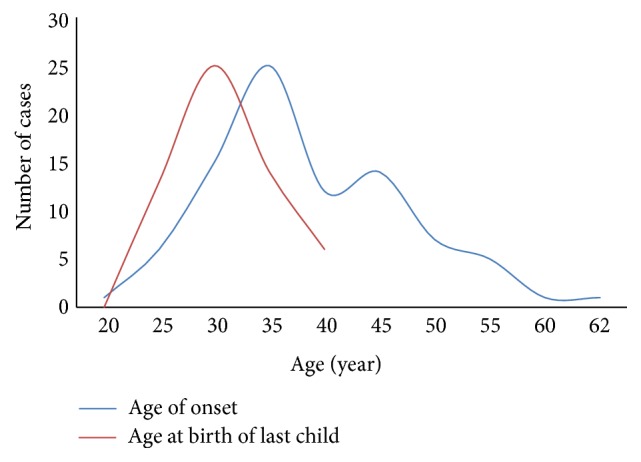
The distribution of age of onset and age at birth of first child. The median age of onset was 34 years, while the median age at birth of last child was 28 years. It seemed that there was a correlation between these two items.

Most patients had unilateral breast disease, with only 8 patients exhibiting bilateral symptoms. Breast mass with or without pain was the most common complaint. The clinical presentations were summarized in Table 2. The median duration between last pregnancy and onset of PDM was 5 years (range 1–35 years), which implied that that reproductive age might relate to PDM to some degree (Figure 1).

### 3.2. Univariate and Multivariate Analysis for the Risk Factors for PDM

To examine the relationship between multiple factors and PDM, a univariate analysis was performed. The results were shown in Table 3. An analysis of the full dataset indicated that overweight/obesity (OR, 3.06; 95% CI, 1.53–6.13; *P* = 0.002), a late onset of menarche (OR, 4.31; 95% CI, 1.95–9.50; *P* < 0.001), having more than one child (OR, 2.75; 95% CI, 1.18–6.41; *P* = 0.019), history of benign breast disease (OR, 4.54; 95% CI, 1.07–10.14; *P* = 0.039), and nipple retraction (OR, 32.76; 95% CI, 4.32–248.61; *P* = 0.001) were statistically significant risk factors for PDM. Late age at first birth (OR, 0.22; 95% CI, 0.09–0.57; *P* = 0.002) had a protective effect for the disease. When stratified at 12 months, duration of breastfeeding was not significantly related to PDM.

All factors in the univariate analysis with *P* values < 0.2 were regarded as candidate predictors for a logistic regression model and a backward variable selection process was used. In the multivariable model (Table 4), overweight/obesity (OR, 1.36; 95% CI, 1.08–1.70; *P* = 0.008) and late onset of menarche (OR, 2.41; 95% CI, 1.38–4.21; *P* = 0.002) were independent predictors of PDM. In contrast, late age at first birth had a protective effect against PDM (OR, 0.18; 95% CI, 0.03–0.98; *P* = 0.048).

### 3.3. Microorganism Infection in PDM

To investigate the role of bacterial infection in the development of PDM, multiple microorganism detection method was employed in this study. Acid-fast bacilli staining was negative in both case and control groups. However, colony formation was found on only one pus specimen plate in bacterial culturing study and two distinct bacterial strains were further identified as* Brevibacterium flavum* and a rare Gram-positive bacterium,* Bacillus cereus,* with 16S rRNA gene sequencing. There was no anaerobic bacterial growth from any sample.

Furthermore, with sequencing of the 16S rRNA gene, we demonstrated that one or more species of bacteria could be detected in all of the patient specimens, but all the control tissues from the benign breast disease were negative on this test. In the case group, a mixture of different bacterial infection was most often observed (38.71%), followed by single bacterial infection of* Pseudomonas* spp. (29.03%) (Table 5).* Enterococcus faecium, Corynebacterium kroppenstedtii*,* Bacillus firmus*,* Sporosarcina*, and* Staphylococcus aureus* were also found in some samples. This result validated the assumption that bacterial infection might be an etiological factor for PDM.

### 3.4. Expression of Proinflammatory Cytokines in PDM

**Figure 2:**
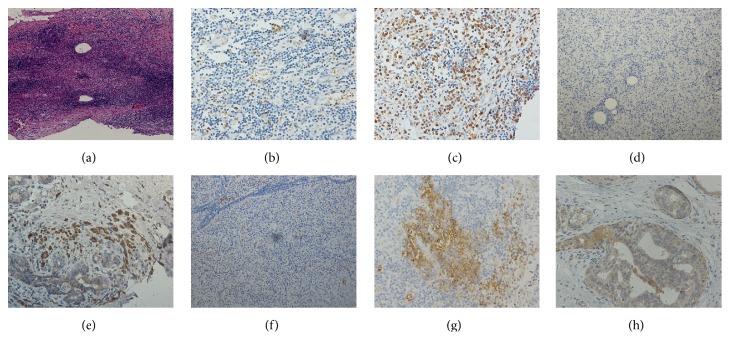
Representative illustrations of the expression of cytokines in PDM and normal breast tissues. (a) Low-power magnification of PDM (hematoxylin and eosin, ×40). (b) Low expression of IFN-*γ* (IHC, ×200). (c) High expression of IFN-*γ* (IHC, ×200). (d) Low expression of IL-12A (IHC, ×100). (e) High expression of IFN-*γ* (IHC, ×200). (f) Low expression of IL-17A (IHC, ×100). (g) High expression of IL-17A (IHC, ×200). (h) The expression of inflammatory cytokines in normal breast ductal epithelium and stromal cells (IHC, ×200).

As bacteria were found in the lesions of PDM, we then test whether the critical proinflammatory cytokines from following immune responses, IFN-*γ*, IL-12A, and IL-17A, were involved in the disease progression with an IHC method (Figure 2). After staining and taking photographs, a scoring evaluation of the density for each cytokine in this IHC photographs was performed and data were summarized in Table 6. As shown in Table 7, the expressions of IFN-*γ* and IL-12A in stromal inflammatory cells of the breast were increased in PDM compared to normal breast tissues. However, there were no significant differences in the expression of IL-17A between these two groups.

**Figure 3:**
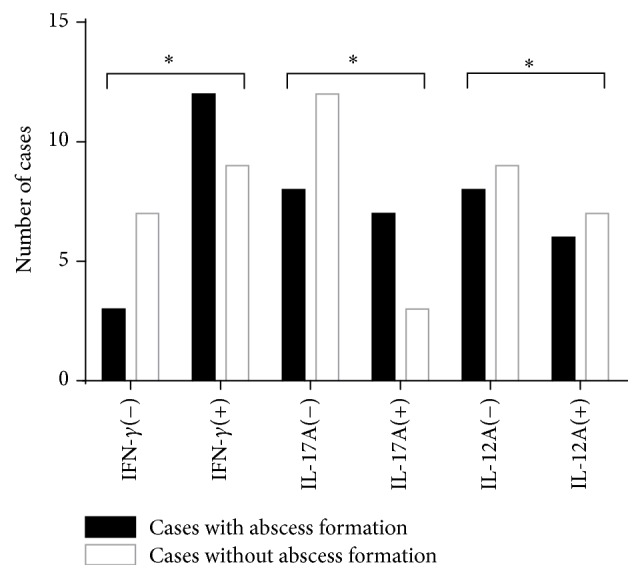
Production of IFN-*γ*, IL-12A, and IL-17A in cases with or without abscess formation. (−): low expression of cytokines; (+): high expression of cytokines. Fifteen patients had lesions with abscess formation, while 16 patients did not have them. More cases with high expressions of IFN-*γ* and IL-17A in stromal inflammatory cells could be found in patients with abscess formation. ^*∗*^*P* > 0.05.

To investigate the cytokines expression profiles at different stages of PDM, the expressions of IFN-*γ*, IL-12A, and IL-17A were then analyzed by grouping with the presence or absence of abscess. Fifteen patients with abscess formation in their lesions and 16 patients without abscess formation were included in this study. As shown in the Figure 3, higher expression levels of IFN-*γ* and IL-17A were found in stromal inflammatory cells from PDM patients with abscess formation than those without abscess formation. These results suggest that proinflammatory cytokines may play an important role in the progression of the disease of PDM, especially the IFN-*γ* and IL-12A. As mentioned above, these two cytokines are key factors involved in the Th1 mediated adaptive immune responses following bacterial infection.

## 4. Discussion

Smoking, obesity, diabetes mellitus, and reproductive factors have been considered as significant risk factors for PDM in previous studies [10, 24]. The results of the present study confirmed that overweight/obesity and the late onset of menarche were independent risk factors for PDM, while late age at first birth had a protective effect. In addition, our results also suggested that nipple retraction was related to PDM. Bacterial infection could be found in all samples of PDM patients by 16S rRNA gene sequencing but not in the samples from the control subjects. Among the infected samples in PDM patients, a mixture of different bacterial strains was the most common condition which could be seen, and then followed by single* Pseudomonas* spp. infection. IFN-*γ* and IL-12A were upregulated in PDM compared to normal breast tissues, but the expression of IL-17A was not significantly different from control samples. Moreover, there are increased expression pattern for both IFN-*γ* and IL-17A in PDM which suggested a following immune response after bacterial infection may contribute the deterioration of PDM.

The incidence of PDM is increasing rapidly during the past decades. Indeed, in our previous investigation, the detection rate of mammary ductal ectasia was 0.24% (147/61,102) [25]. However, the etiology of PDM is unclear yet, mostly due to lack of conclusive evidences from previous study. Although some studies suggest smoking is a risk factor for PDM [11, 12], smoking was not found to be closely related to PDM in the present study. One of the plausible reasons might be the low prevalence of smoking among Chinese women [26].

Obesity is related to low-grade chronic inflammation, which can disrupt immune function [27, 28]. Furthermore, obesity can directly influence local mammary estrogen and inflammation [29]. Gollapalli et al. [8] reported that obesity was a risk factor for breast abscess. Bharat et al. [13] found that higher BMI values were also associated with nonpuerperal abscess. They also reported similar results in cases of granulomatous lobular mastitis. In PDM, however, they did not find a significant effect of obesity on patients when compared with health controls [30]. In our study, data demonstrated obesity is another risk factor for PDM. Obesity may directly disrupt the local immune functioning of the breast and exacerbate the development of PDM.

The relationship between reproductive factors and PDM is very interesting. Inconsistent with the finding reported previously from literature in which parity was not found to be associated with the risk for duct ectasia [31], our results suggest that reproductive factors were related to PDM. We also found late onset of menarche was a significant risk factor for PDM while increased age at first birth was a protective factor. The changes of serum prolactin and lactation in patients may be responsible for the above association. The exact mechanism underlying this relationship is still unknown; future investigation focusing on theses hormones is warranted.

The relationship between nipple retraction and PDM has been well-documented [32]. Nipple retraction could induce breast deformation and influence the process of lactation. Furthermore, it also can obstruct drainage and lead to an accumulation of massive substances in ducts, which is termed mastitis [33]. In this study, we confirmed that nipple retraction was a risk factor for PDM. The prevalence rate of nipple retraction is approximately 3.26% according to previous report [34]. But, over one quarter of PDM patients experienced nipple retraction in our study.

Bacterial infection is considered to be another possible etiology of PDM, although it has been a long-standing controversial problem. Al Benwan et al. [15] found that* S. aureus* was the predominant pathogen, followed by* Bacteroides* spp.*, Anaerobic strep.*, and* P. aeruginosa.* However, the relationship between bacteria and PDM was not confirmed in other report [31]. Here, we provided more evidence to confirm for this assumption. Inconsistent with previous report [35], we did not find that either* S. aureus* was the most common bacterium in PDM or anaerobic bacteria was associated with this disease based on our current data. Interestingly, the bacteria identified here were those microorganisms which are often related to community-acquired infection, including* Pseudomonas* spp. and* S. aureus*. Collectively, these finding suggests that bacterial infection may only be acting as an induction factor and the immune system which are following the bacterial infection might play a major role in the development of PDM. However, more study is needed for the future to determine the common route in which the bacterial infection and the following immune response converge.

IFN-*γ* and IL-12A are characteristic Th1 cytokines [36]. Th1 cells are important for the eradication of invading pathogens, including bacteria, parasites, yeast, and viruses [37]. In this study, the expressions of IFN-*γ* and IL-12A were upregulated in breast stromal inflammatory cells of PDM patients. Recently, a lineage of CD4^+^ T cells producing IL-17A was described and accordingly named as Th17 cells. IL-17A has been shown to be important for host defense against pathogens [38]. However, in our study, there was no significant difference in IL-17A expression between the two groups. As IFN-*γ* and IL-12A were detected in PDM tissue samples, there is a rationale to conclude that the Th1 immune response plays a role in this disease. The mechanism by which these cytokines contribute to the course of disease is still unknown and needs further investigation.

The strengths of the present study include the following: (a) a relatively large population of patients was included; (b) the assessment of tissue was done by two histopathologists experienced in breast inflammation. However, our study also had several limitations. First, this was a nonmatched case control study and there might be some uncontrolled biases which may influence the credibility of the results. Second, there was an issue with missing data, especially the data of reproductive factors, which may also lead to the bias of results. Finally, we have not investigated the mechanism by which IFN-*γ* and IL-12A participated in the course of the disease.

## 5. Conclusions

The results of the present study indicated that overweight/obesity and later onset of menarche are independent risk factors for PDM. In contrast, age of first birth >27 years is a protective factor for PDM. The importance of these reproductive factors to the risk of PDM has not been previously reported and will provide important novel information to broaden the avenue of investigating the etiology of PDM in the future. We also confirm that bacterial infection with a mixture of different bacterial species is a closely associated with the pathogenesis of PDM. Moreover, proinflammatory cytokines, IFN-*γ* and IL-12A, were also shown to be associated with PDM, which indicates that the Th1 immune response may closely relate to this disease. Therefore, PDM could be an inflammatory disease related to bacterial infection and consequent immune responses with increased cytokines. As far as we know, this is first report about the possible immune responses following bacterial infection which is involved in development of PDM disease, and more research is needed for the future to further investigate the pathogenesis of PDM on the mechanism by which bacterial infection and consequent immune responses affect the progression of the disease.

## Figures and Tables

**Table 1:** Demographic characteristics in case and control groups.

Variable	Cases (*n* = 87)	Controls (*n* = 87)	^*∗*^ *P*
Marriage			
Yes	82 (94.25)	86 (98.85)	0.211
No	5 (5.75)	1 (1.15)	
Menopause			
Yes	6 (6.90)	0 (0)	0.029
No	81 (93.10)	87 (100)	
Hypertension			
Yes	5 (5.75)	0 (0)	0.059
No	82 (94.25)	87 (100)	
Diabetes mellitus			
Yes	5 (5.75)	0 (0)	0.059
No	82 (94.25)	87 (100)	
Heart diseases			
Yes	4 (4.60)	0 (0)	0.121
No	83 (95.40)	87 (100)	
Passive smoking			
Yes	23 (46.94)	16 (35.56)	0.263
No	26 (53.06)	29 (64.44)	
History of allergies			
Yes	9 (10.34)	7 (8.05)	0.670
No	78 (89.66)	80 (91.95)	

*Note.* Data are number (%) of patients.

^*∗*^ *P* values were determined with the chi-square test.

**Table 2:** Clinical characteristics and presentations of patients with PDM.

Characteristic	Value
Mean size of mass (cm)	3.91 ± 2.57
Affected sides	
Right	34 (39.08)
Left	45 (51.72)
Bilateral	8 (9.20)
Quadrant(s)	
Upper-outer	20 (22.99)
Lower-outer	8 (9.20)
Upper-inner	9 (10.34)
Lower-inner	9 (10.34)
Two or more quadrants	15 (17.24)
Subareolar region	26 (29.89)
Clinical presentation	
Mass without pain	63 (72.41)
Mass with pain	15 (17.24)
Erythroswelling	31 (35.63)
Nipple discharge	6 (6.90)
Sinus	12 (13.79)
Nipple retraction	24 (27.59)
Biopsy methods	
Incisional or excisional biopsy	24 (27.59)
Vacuum-assisted biopsy	63 (72.41)

*Note.* Data are number (%) of patients, unless otherwise indicated.

**Table 3:** Univariate analysis of selected risk factors for PDM^*∗*^.

Variable	Cases (*n* = 87)	Controls (*n* = 87)	OR (95% CI)	*P*
Age (years)				
<35	48 (55.17)	56 (64.37)	1.0 (Ref)	0.217
≥35	39 (44.83)	31 (35.63)	1.47 (0.80–2.70)	
BMI (kg/m^2^)				
<24	34 (39.08)	65 (74.71)	1.0 (Ref)	0.002
≥24	34 (39.08)	20 (22.99)	3.25 (1.63–6.48)	
Unknown	19 (21.84)	2 (2.30)		
Age at menarche (years)				
≤14	57 (65.52)	74 (85.06)	1.0 (Ref)	0.001
>14	30 (34.48)	10 (11.49)	3.90 (1.76–8.62)	
Unknown	0 (0.00)	3 (3.45)		
Parity				
≤1	64 (73.56)	72 (82.76)	1.0 (Ref)	0.019
>1	22 (25.29)	9 (10.34)	2.75 (1.18–6.41)	
Unknown	1 (1.15)	6 (6.90)		
Miscarriages				
No	23 (26.44)	25 (28.74)	1.0 (Ref)	0.470
Yes	25 (28.74)	36 (41.38)	0.76 (0.35–1.62)	
Unknown	39 (44.82)	26 (29.88)		
Age at birth of first child (years)				
≤27	34 (39.08)	26 (29.89)	1.0 (Ref)	0.001
>27	8 (9.20)	29 (33.33)	0.21 (0.08–0.54)	
Unknown	45 (51.72)	32 (36.78)		
Duration of breastfeeding (months)				
≤12	34 (39.08)	42 (48.28)	1.0 (Ref)	0.688
>12	36 (41.38)	39 (44.83)	1.14 (0.60–2.16)	
Unknown	17 (19.54)	6 (6.89)		
History of benign breast disease				
Yes	6 (6.90)	3 (3.45)	4.54 (1.07–19.14)	0.039
No	37 (42.53)	84 (95.55)	1.0 (Ref)	
Unknown	44 (50.57)	0 (0.00)		
Galactostasis				
Yes	23 (26.44)	32 (36.78)	0.86 (0.44–1.70)	0.670
No	40 (45.98)	48 (55.17)	1.0 (Ref)	
Unknown	24 (27.58)	7 (8.05)		
Nipple retraction				
Yes	24 (27.59)	1 (1.15)	32.76 (4.32–248.61)	0.001
No	63 (72.41)	86 (98.85)	1.0 (Ref)	

*Note.* Data are number (%) of patients. BMI: body mass index, calculated as weight in kilograms divided by the square of height in meters. OR: odd ratio. CI: confidence interval.

^*∗*^Using binary logistic regression.

**Table 4:** Multivariate logistic regression analysis of the associations between various factors and PDM.

Variable	B	S.E.	Wald	OR	95% CI	*P*
Overweight/obesity	0.31	0.12	7.00	1.36	1.08–1.70	0.008
Age at first birth	−1.70	0.86	3.92	0.18	0.03–0.98	0.048
Age at menarche	0.88	0.29	9.53	2.41	1.38–4.21	0.002

**Table 5:** Multiple bacteria could be detected in PDM patients.

Bacteria	*N* (%) (*n* = 31)
Mixture of different bacteria	12 (38.71)
*Pseudomonas*	
*Pseudomonas aeruginosa*	5 (16.13)
*Uncultivated Pseudomonas*	2 (6.45)
*Pseudomonas delhiensis*	1 (3.23)
*Pseudomonas otitidis*	1 (3.23)
*Enterococcus faecium*	2 (6.45)
*Corynebacterium kroppenstedtii*	1 (3.23)
*Bacillus firmus*	1 (3.23)
*Sporosarcina*	1 (3.23)
*Staphylococcus aureus*	1 (3.23)
*Uncultured bacterium clone*	4 (12.90)

**Table 6:** The expressions of IFN-*γ*, IL-12A, and IL-17A in PDM compared with normal breast tissues.

Score	IFN-*γ*		IL-12A		IL-17A	
	Case	Control	Case	Control	Case	Control
0	2	1	0	2	1	2
1	2	3	0	3	10	1
2	5	13	11	12	8	10
3	1	2	6	2	1	4
4	8	3	7	1	10	3
6	9	0	4	1	0	2
8	0	0	0	0	0	0
9	4	0	2	1	0	0
Absent	0	0	1^#^	0	1^#^	0

^#^Samples were lost in the process of IHC.

**Table 7:** The correlationship of IFN-*γ*, IL-12A, and IL-17A expression in PDM compared with normal breast tissues.

Cytokines	Case	Control	*P* ^*∗*^
Low expression of IFN-*γ*^#^	10	19	<0.001
High expression of IFN-*γ*	21	3	
Low expression of IL-12A	13	19	0.022
High expression of IL-12A	17	3	
Low expression of IL-17A	20	17	0.404
High expression of IL-17A	10	5	

^*∗*^ *P* values were determined with the chi-square test.

^#^Low expression was defined as the total score ≤ 3, and others were defined as high expression.
